# Strip-Pattern-Spheres Self-Assembled from Polypeptide-Based Polymer Mixtures: Structure and Defect Features

**DOI:** 10.1038/srep29796

**Published:** 2016-07-15

**Authors:** Xingyu Zhu, Zhou Guan, Jiaping Lin, Chunhua Cai

**Affiliations:** 1 Shanghai Key Laboratory of Advanced Polymeric Materials, State Key Laboratory of Bioreactor Engineering, Key Laboratory for Ultrafine Materials of Ministry of Education, School of Materials Science and Engineering, East China University of Science and Technology, Shanghai 200237, China

## Abstract

We found that poly(γ-benzyl-L-glutamate)-*block*-poly(ethylene glycol) (PBLG-*b*-PEG) rod-coil block copolymers and polystyrene (PS) homopolymers can cooperatively self-assemble into nano-spheres with striped patterns on their surfaces (strip-pattern-spheres) in aqueous solution. With assistance of dissipative particle dynamics simulation, it is discovered that the PS homopolymers form a spherical template core and the PBLG-*b*-PEG block copolymers assemble into striped patterns on the spherical surface. The hydrophobic PBLG rods are packed orderly in the strips, while the hydrophilic PEG blocks stabilize the strip-pattern-spheres in solution. Defects such as dislocations and disclinations can be observed in the striped patterns. Self-assembling temperature and sphere radius are found to affect defect densities in the striped patterns. A possible mechanism is proposed to illustrate how PBLG-*b*-PEG and PS cooperatively self-assemble into hierarchical spheres with striped patterns on surfaces.

Self-assembly of polymer mixtures is an effective method to obtain hierarchical structures with various morphologies^1^,^2^,^3^,^4^. Hierarchical structures have ordered features with various levels of microscopic length scales^5^,^6^. For example, Ikkala and ten Brinke reported the self-assembly of polystyrene-*block*-poly(4-vinylpyridine) (PS-*b*-P4VP) and pentadecylphenol (PDP) mixtures. The PDP compounds are attached to P4VP blocks through hydrogen bonds between phenol and pyridine groups to form supramolecules. The supramolecules can self-assemble into hierarchical microstructures such as perpendicularly packed lamella-*in*-lamella^7^. Through understanding the formation mechanism of the hierarchical structures, novel materials with specific functions can be designed and fabricated^8^,^9^,^10^,^11^,^12^. Therefore, synthesis of defined hierarchical structures by self-assembly of polymer mixtures attracts great attention recently^13^,^14^,^15^.

In a preliminary work, we have discovered that rod-coil block copolymer poly(γ-benzyl-L-glutamate)-*block*-poly(ethylene glycol) (PBLG-*b*-PEG) and rigid homopolymer poly(γ-benzyl-L-glutamate) (PBLG) could cooperatively self-assemble into super-helical structures with uniform chirality and screw-pitch in solution^16^,^17^,^18^. In the self-assembled super-helices, the homo-PBLG formed a fiber-like bundle while the block copolymers composed a screwed shell. Replacing the rigid homo-PBLG by homo-PS, the mixtures of PBLG-*b*-PEG and homo-PS self-assembled into spherical structures with striped patterns on their surfaces (strip-pattern-spheres) which are like “wool balls”. In this case, the PS homopolymers may form spherical templates and then the PBLG-*b*-PEG block copolymers self-assemble into striped patterns on the template surfaces. Recently, through further examination of these self-assembled structures, we discovered that geometrical defects exist in the strip-pattern-spheres.

Striped patterns with defects exist in diverse systems varying from microscopic structures up to macroscopic objects^19^. The existence of defects in striped patterns is an important natural geometrical phenomenon, and many essential mechanisms of this phenomenon are still unrevealed. Distributions and dynamics of defects in two-dimensional (2D) striped patterns have been studied by both simulation and experiment methods^20^,^21^,^22^,^23^,^24^,^25^,^26^,^27^,^28^. Moreover, when the striped patterns appear on the spherical or curved surfaces, new features come out due to the restriction of topological condition^29^,^30^,^31^,^32^,^33^,^34^,^35^,^36^,^37^,^38^,^39^,^40^,^41^,^42^. According to Poincaré-Hopf theorem^43^, the total disclination charge of a geometrical structure follows the equation 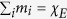, in which *i* represents defect number, *m*_*i*_ is the value of *i*-th disclination charge, χ_*E*_ is the Euler characteristic. For spherical structure, the total charge is +2, which means there are defects which cannot be annihilated on spherical surface. The studies about striped patterns with defect structures on spherical surfaces are still limited due to the challenge to conduct proper experiments.

In addition, so far most of the reported striped patterns are formed by coil-coil block copolymers on planar surface^44^,^45^,^46^,^47^. Few studies of defect features in striped patterns formed by rod-coil copolymers have been reported as far as we know. Rigid chains tend to form ordered structure due to the driving forces of enthalpy^48^. Introducing rigid chains into polymers provides new influences for the formation of striped patterns. Herein, it is fundamentally interesting to investigate striped pattern structures self-assembled from rod-coil copolymers. In fact, some natural structures, such as the arrangement of proteins with features of rigid molecules on biological membrane, show similar physical characteristics to the striped patterns on spherical surfaces^49^,^50^,^51^. Exploring the ordered packing of rigid polymers in striped patterns may make a contribution to understanding the biological patterns in nature.

In this work, we investigated the self-assembly behavior of PBLG-*b*-PEG/PS mixtures in aqueous solution. Nano-spheres with striped patterns on its surfaces were found. In the self-assembled strip-pattern-spheres, defects such as dislocations, +1/2 disclinations and −1/2 disclinations were observed. Effects of self-assembling temperature (*T*) and sphere radius (*R*_h_) were investigated experimentally to reveal the evolution of defect densities (area-average number of defects). Simulations based on dissipative particle dynamics (DPD) method were carried out, providing more detailed information about the self-assemblies, which could not be obtained experimentally because of the limitation of characterization methods. Influencing parameters such as interaction between rod and solvent (*a*_RS_) and sphere radius (*R*) were further explored to verify the results theoretically. Finally, a possible mechanism was proposed to illustrate the self-assembly behavior of PBLG-*b*-PEG/PS mixtures.

## Results

For the preparation of strip-pattern-spheres, PBLG-*b*-PEG/PS were firstly dissolved in mixture solvent of THF/DMF (tetrahydrofuran/*N,N′*-dimethylformamide, 1/1, v/v). When selective solvent (water) was added to the initial solution, PS homopolymers could firstly aggregate into spheres due to the lower CWC (critical water content) compared with that of PBLG-*b*-PEG block copolymers (for details, see following section). Further addition of water gave rise to the aggregation of PBLG-*b*-PEG onto the surfaces of PS cores, forming striped patterns on the spheres. The spherical aggregates with striped patterns are stable after dialysis in water. Store temperature shows no obvious effect on the morphologies of the aggregates (details are shown in Supporting Information, Figure S7).

**Figure 1:**
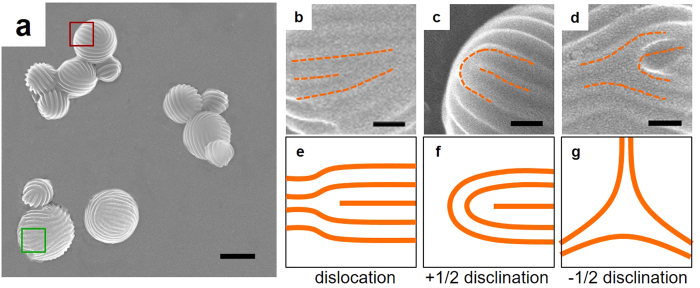
Striped patterns with defect features. (**a**) SEM images of PBLG_12100_-*b*-PEG_5000_ /PS_19400_ aggregates. (**b,e**) dislocation; (**c,f**) +1/2 disclination; (**d,g**) −1/2 disclination. (**b–d**) are SEM images from experiments. (**e–g**) are schematic illustrations. (**b,c**) show the enlarged images of green and red squares in (**a**), respectively. Scale bars: 400 nm for (**a**) and 100 nm for (**b–d**).

Figure 1a shows the typical SEM image of strip-pattern-spheres self-assembled from PBLG_12100_-*b*-PEG_5000_/PS_19400_ (the subscripts denote the number-average molecular weight) mixture systems. The self-assembly was performed at 20 °C, and the weight fraction of PBLG-*b*-PEG (*f*_PBLG-*b*-PEG_) in the polymer mixture is 0.8. On the surfaces of these spheres, we can see striped patterns with defect features. Three types of defects can be identified in our experiments: dislocation, +1/2 disclination, and −1/2 disclination. Dislocation and + 1/2 disclination are marked in the SEM image by green and red squares (Fig. 1a), respectively. The enlarged images of the dislocation and +1/2 disclination are further presented in Fig. 1b,c, respectively. The −1/2 disclination presented in Fig. 1d is rarely observed in this system. Figure 1e–g display schematic illustrations of these typical topological defects. Details about the identification of the defects are shown in Supporting Information, Section 3.1.

**Figure 2:**
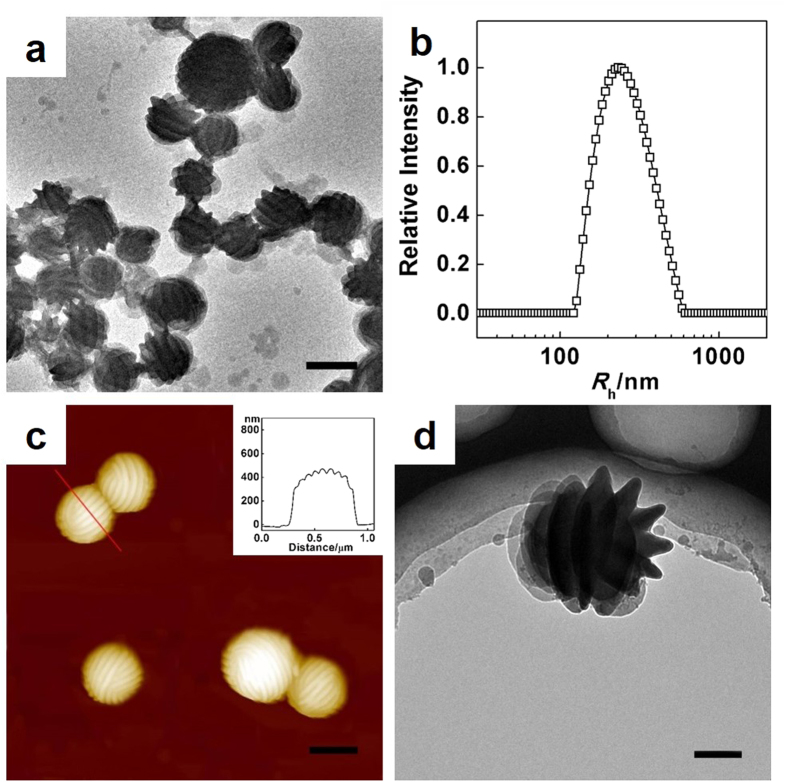
Typical strip-pattern-spheres observed in experiments. (**a**) TEM, (**c**) AFM, and (**d**) cryo-TEM images of PBLG_12100_-*b*-PEG_5000_/PS_19400_ aggregates. (**b**) *R*_h_ distribution of the aggregates in solution obtained by DLS. The inset in (**c**) shows the height profile along the red line in (**c**). Scale bars: 400 nm for (**a**,**c**); 200 nm for (**d**).

The structure of the strip-pattern-spheres was further confirmed by TEM, DLS, AFM, and cryo-TEM characterization. As shown by TEM image (Fig. 2a), the diameter of the self-assembled strip-pattern-spheres ranges from *ca.* 200 nm to *ca.* 500 nm. Figure 2b shows DLS result of the aggregates in water solution, which reveals that the average hydrodynamic radius (*R*_h_) of the spherical aggregates is 283 nm. AFM image (Fig. 2c) and the height profile (the inset of Fig. 2c) show similar morphology results. Cryo-TEM image (Fig. 2d) shows the spherical structures with striped patterns which are the same as the aggregates shown by SEM and TEM, indicating drying process has no effects on the structure of strip-pattern-spheres obtained in this system. In addition, as revealed by the SEM and AFM images, widths of the strips are *ca.* 45 nm and the striped patterns have almost uniform pitch size (*ca.* 69 nm).

**Figure 3:**
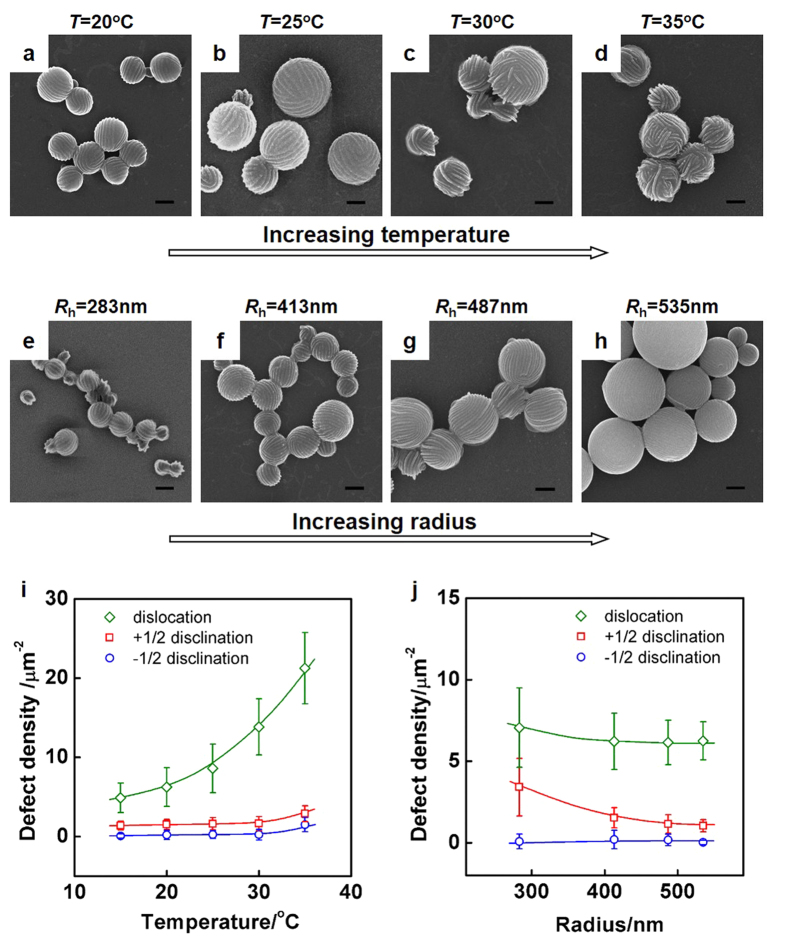
The effect of temperatures (*T*) and radii (*R*_h_) on defect densities in experiments. SEM images of PBLG-*b*-PEG/PS aggregates self-assembled at various temperatures (*T*) and radii (*R*_h_): (**a**) *T* = 20 °C; (**b**) *T* = 25 °C; (**c**) *T* = 30 °C; (**d**) *T* = 35 °C; (**e**) *R*_h_ = 283 nm (*f*_PBLG-*b*-PEG_ = 0.8); (**f**) *R*_h_ = 413 nm (*f*_PBLG-*b*-PEG_ = 0.6); (**g**) *R*_h_ = 487 nm (*f*_PBLG-*b*-PEG_ = 0.4); (**h**) *R*_h_ = 535 nm (*f*_PBLG-*b*-PEG_ = 0.2). Scale bars: 400 nm. (**i**) Statistic defect densities as a function of temperature. (**j**) Statistic defect densities as a function of sphere radius. *R*_h_ data were obtained by DLS measurements.

To deepen the understanding of the formation of defect features in the strip-pattern-spheres, we further examined the effects of self-assembling temperature (*T*) and sphere radius (*R*_h_). Figure 3a–d show the SEM images of self-assembled strip-pattern-spheres formed at temperatures of 20 °C, 25 °C, 30 °C, and 35 °C, respectively. It was found that the striped patterns on spheres become less regular at higher temperatures. To quantitatively evaluate the dependence of the defect structure on temperature, the numbers of dislocation and disclination defects were counted based on large number of SEM images (for details of statistics method, refer to Figure S4 in Supporting Information). It should be noted that SEM images only reflect the top side morphology of the sample. Therefore, for each ball, approximately 3/8 of the total area on the spherical surface can be observed (the defect densities in experiment were calculated based on this area). Figure 3i shows the dependence of area-average defect number (defect density) on self-assembling temperature. The density of dislocations increases obviously with increasing temperature while the densities of both +1/2 and −1/2 disclinations increase slightly. According to the Poincaré-Hopf theorem^43^, the total disclination charge on the spherical surface should be +2, thus disclinations with charge of +1/2 and −1/2 vary synchronously to maintain the total charge on the spherical surface. The temperature effect can be rationalized by considering the interplay of entropy and enthalpy in the self-assembly system. When temperature is lower, the entropy could have less contribution to the free energy. Under such circumstance, the formation of pattern structure is dominated by enthalpy^52^,^53^. The PBLG blocks tend to be orderly packed due to stronger interactions between PBLG blocks and regular patterns with fewer defects can be observed. As temperature increases, entropy plays more important roles. This could lead to less orderly packed chains. As a result, less regular patterns with more dislocation defects are observed. Although the temperature has significant effect on the defect densities, its influence on the width of strips and pitch of the patterns is negligible. Both the strip width and pitch size almost remain the same in the temperature range employed (for details, see Figure S5).

In addition to temperature, we examined the influence of sphere radius on the defects of striped patterns. The core of the sphere consists of hydrophobic PS homopolymers, which determines the radius of the aggregates. Therefore, by changing the weight fraction of PBLG-*b*-PEG (*f*_PBLG-*b*-PEG_) in the PBLG-*b*-PEG/PS mixtures, we can manipulate the radius of self-assembled aggregates. Figure 3e–h show that the radius of the sphere increases with decreasing the weight fraction of PBLG-*b*-PEG block copolymers in the mixtures. The average sphere radius *R*_h_ for each composition was obtained from DLS measurement. The dependence of defect density on the sphere radius is presented in Fig. 3j. The results show that defect density of +1/2 disclinations decreases with increasing radius. This is mainly because the number of +1/2 disclinations is almost unchanged while the surface area enlarges. Since the number of −1/2 disclinations remains nearly zero, the density of −1/2 disclinations does not vary obviously as the radius changes. From Fig. 3j, we also see that the density of dislocations decreases slightly with increasing radius. This indicates that the increase of surface area causes increasing random dislocations.

Additionally, the effects of some other factors, such as molecular weight of PBLG blocks and chain rigidity of block copolymers, on the self-assembled strip-pattern-spheres have been investigated. The studies of influences of molecular weight of PBLG blocks show that most of the block copolymers aggregate into small spheres instead of assembling on the surface of PS core as molecular weight of PBLG blocks is higher (Figure S10 in Supporting Information). The chain rigidity of block copolymers is another factor influencing the formation of strip-pattern-spheres. Replacing rod-coil block copolymer PBLG-*b*-PEG by coil-coil block copolymer PS-*b*-PEG, only spheres without pattern structure can be observed (details are shown in Supporting Information, Figure S10c in Section 3.8).

**Figure 4:**
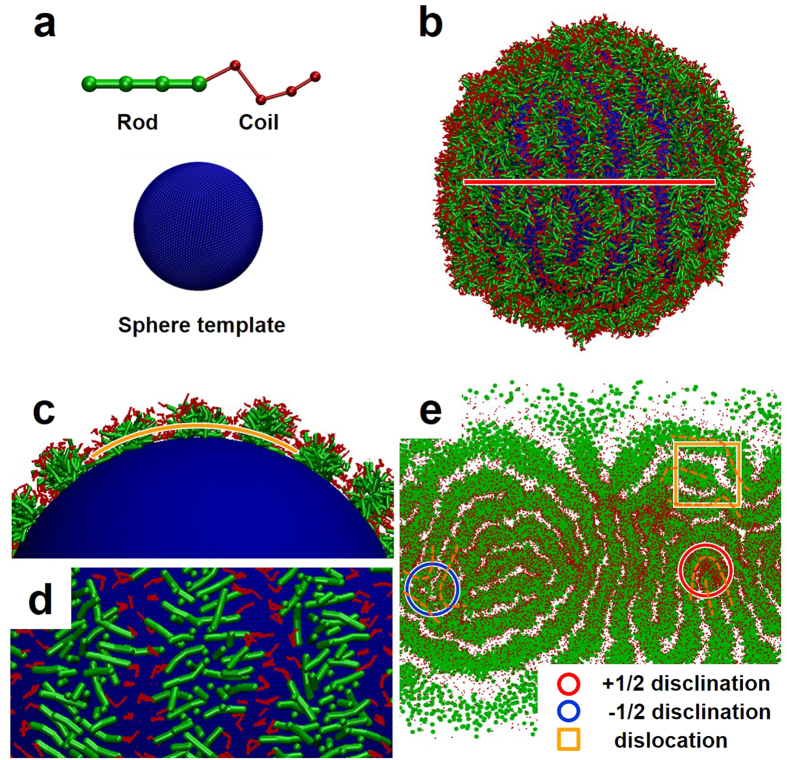
Typical strip-pattern-spheres obtained from simulations. (**a**) Schematic DPD model of rod-coil block copolymer and spherical template. Rod, coil blocks, and spherical substrate are colored green, red, and blue, respectively. (**b**) Simulation prediction of striped patterns formed by rod-coil block copolymers on the spherical surface. (**c**) The cross section image of the spherical structure along the red line in (**b**). (**d**) The cross section image of the strips along the yellow line in (**c**). (**e**) 2D flat projection of (**b**). In (**e**), +1/2 disclination is marked with red circle, −1/2 disclination is marked with blue circle and dislocation is marked with yellow rectangle. Solvents are not shown in these Figures.

The limitations of current experimental technique could hinder our deep understanding of the mechanism underlying the striped pattern formation on the surfaces of self-assembled spheres. To address this challenge, dissipative particle dynamics (DPD) simulations were conducted to explore the inherent structure of the aggregates. Inferring from dynamic process of the sphere formation, PS homopolymers firstly aggregate into spherical cores and then the block copolymers assemble on the surfaces to form striped patterns (the formation process is proved by simulation as well, which is shown in Supporting Information, Section 2.3). In simulations, the process was simplified to focus on the ordered packing process of block copolymers. A fixed template ball was applied to represent the self-assembled PS core. To simulate the PBLG-*b*-PEG/PS mixture system, a model containing one template ball (23522 beads for *R* = 10 *r*_c_, this value may change with various radii of spheres, *r*_c_ is cutoff distance) and R_4_C_4_ rod-coil block copolymers, whose amount may vary with the sphere radius, were constructed (Fig. 4a). The hydrophobic R segments are rigid mimicking the PBLG block, and the hydrophilic C segments are flexible mimicking PEG blocks. The DPD simulation under NVT ensemble was performed in a periodic boundary conditioned box. Corresponding to the experiments, the interaction parameters between each component are *a*_RC_ = 80, *a*_RS_ = 80, *a*_RP_ = 25, *a*_CS_ = 30, *a*_CP_ = 25, and *a*_PS_ = 120, where R, C, S, and P represent rod blocks, coil blocks, solvents, and the beads constructing the template ball, respectively (simulation details are shown in Supporting Information, Figure S1). Revealed by the simulation results, the assembled structure remains unchanged after 10000 τ (τ is the time unit), which indicates that the simulation time shows negligible effect on defect feature after 10000 τ. In practice, all of the structures in simulations were obtained at 20000 τ to ensure equilibrium states have been reached. Figure 4b presents the obtained simulation results. As can be seen, strip-pattern-sphere was successfully reproduced (the radius of the template ball, modeled by 75002 beads, is 18, and the number of copolymers is 5816). For the striped patterns, the hydrophobic R rods are packed orderly to form the inner parts of the strips, and the hydrophilic C blocks extend into the solution to stabilize the structures. Figure 4c shows the cross section image of the simulated structure, which reveals that the hydrophobic rods lie closely on the surface of the template sphere. The packing of R rods is in a way that the free ends are in center of the strips and the ends connected to C segments are close to the shell. Figure 4d shows that the strips are composed of rods which order perpendicular to the strip axis. Taking this packing mode in consideration, *i.e.* the pitch size could be approximately twice the block copolymer length, we calculated the lengths of copolymer blocks, which are 8.25 nm for rigid PBLG blocks and 39.2 nm for fully extended PEG blocks, respectively. In experiments, the observed pitch size is *ca.* 69 nm, which can be roughly equal to twice the length of block copolymers as the PEG chains are usually flexible and not fully extended. This calculation suggests that the simulation results are reasonable. Details about the calculations are provided in Supporting information, Section 3.3. In order to get the total number of defects on the entire spherical surface, we projected the 3D simulated morphology to 2D flat (Fig. 4e). The +1/2 disclination (red circle), −1/2 disclination (blue circle) and dislocation (yellow rectangle) can be identified directly in the two-dimensional flat projection of the simulation structure. From the above results, we can see that the simulations well reproduced the experimental results. It further provided information which can hardly be obtained by experimental characterizations. For example, the ordered manner of polymer chains and the entire striped patterns on the spherical surface.

**Figure 5:**
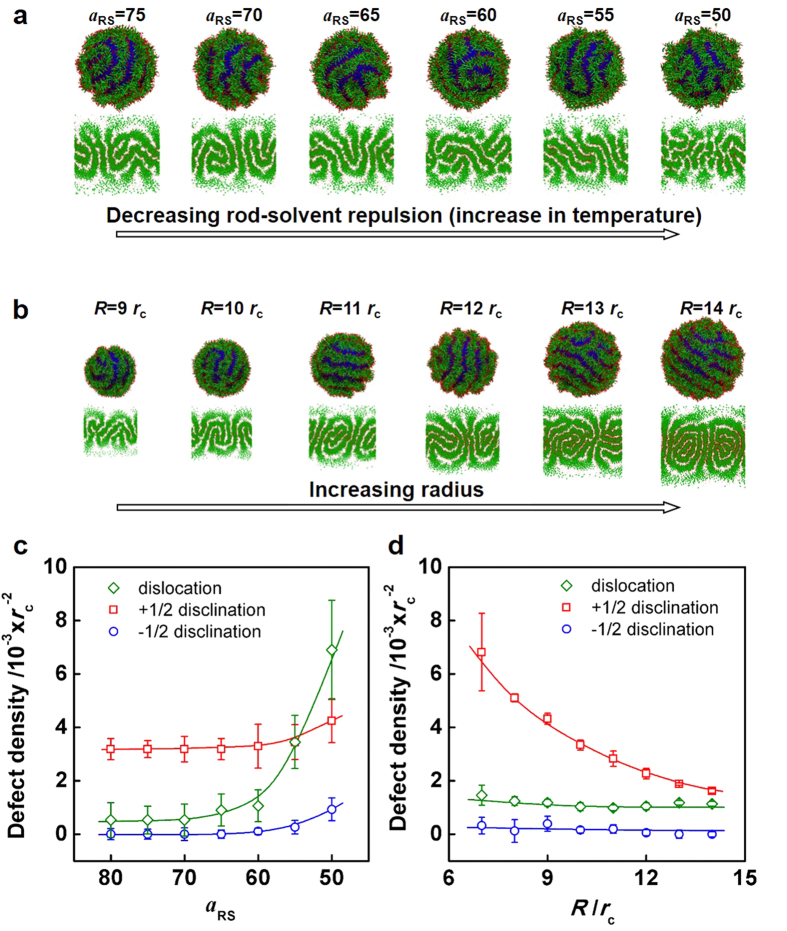
The effect of rod-solvent parameter (*a*_RS_) and sphere radius (*R*) on defect densities in simulations. Simulation prediction and flat projection of strip-pattern-spheres with various rod-solvent parameter (*a*_RS_) and sphere radius (*R*): (**a**) *a*_RS_ = 75, 70, 65, 60, 55, 50, *R* = 10 *r*_c_; (**b**) *a*_RS_ = 80, *R* = 9, 10, 11, 12, 13, 14 *r*_c_. (**c**) Statistic defect densities as a function of rod-solvent parameter (*a*_RS_). (**d**) Statistic defect densities as a function of sphere radius (*R*).

From the experiments, we learned that temperature has marked influence on the defect densities of formed striped patterns. Higher temperature leads to relatively stronger attraction between PBLG segments and solvents. Because the rod-solvent interaction parameter (*a*_RS_) is associated with the repulsion between PBLG rods and solvents, the parameter (*a*_RS_) is relatively smaller at higher temperature^53^. To simulate the changes of temperature, we vary the rod-solvent interaction parameter (*a*_RS_) from 80 to 50, corresponding to the increase in temperature. Figure 5a presents strip-pattern-spheres with various *a*_RS_. The strips are less continuous and the patterns are less regular when the *a*_RS_ becomes smaller. As a comparison to experiment, simulation prediction of the defect densities as a function of temperature-related *a*_RS_ is presented (Fig. 5c). With *a*_RS_ decreasing from 80 to 50 (corresponding to the increase in temperature), the density of dislocations increases obviously and the densities of both disclinations increase slightly. The sum of disclinations charge maintains +2 on one sphere, which accords with Poincaré-Hopf theorem^43^. The simulation result of the *a*_RS_ effect shows a similar tendency with the temperature-dependent results from experiments, which further verifies the experiments. In addition, similar to experimental observations at various temperatures, *a*_RS_ shows negligible effect on the strip width and pitch size (Figure S5).

As revealed by the experimental results, the radii of the spherical aggregates have influences on striped patterns formed by the rod-coil block copolymers. To explore the effect of the sphere radius, strip-pattern-spheres were simulated for the system with various radii (*R*) of the template spheres (It should be noted that “*R*” is the radius of the sphere in simulations and “*R*_h_” is the hydrodynamic radius of the spherical structure self-assembled in experiments). In the simulation, the amount of copolymers varied synchronously with the sphere radius, corresponding to the experimental studies. This could guarantee constant thickness of the block copolymer layer. Statistic results of defect densities were acquired based on the 2D projections of the striped patterns. Similar to the experimental results (Fig. 3j), the density of +1/2 disclinations decreases while the density of −1/2 disclinations remain almost constant with increasing radius (Fig. 5d). The density of dislocations decreases slightly, which is in line with experimental results. Here, it should be noted that the values of dislocation density in simulations are relatively smaller than those obtained from experiments. This could be attributed to the fact that the radius of spheres in simulations is relatively smaller, leading to fewer random dislocation. Additionally, as revealed by simulations, the width of strips and pitch of the patterns remain almost the same when the radii of the template spheres (*R*) increase from 9 to 14 *r*_c_ (Fig. 5b and Figure S5). The simulation results of the radius effect further verify the experiments and prove the conclusion theoretically.

**Figure 6:**
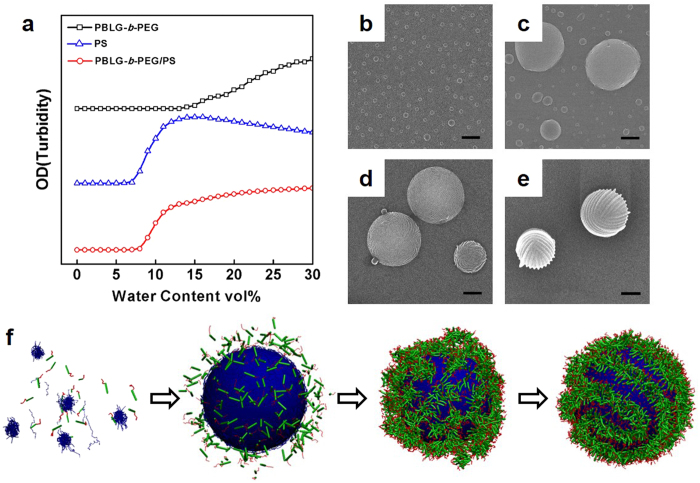
Formation mechanism of the strip-pattern-spheres. (**a**) Turbidity (optical density) curves of PBLG-*b*-PEG block copolymers, PS homopolymers, and PBLG-*b*-PEG/PS polymer mixtures as a function of added water content to the solutions. The initial solvent is THF/DMF = 1/1 in volume. SEM images of PBLG-*b*-PEG/PS aggregates with various added water content: (**b**) 9.0 vol%, (**c**) 10.0 vol%, (**d**) 15.0 vol%. (**e**) SEM images of PBLG-*b*-PEG/PS aggregates after dialysis. (**f**) Schematic illustration of formation process of strip-pattern-spheres. Scale bars: 400 nm.

To better understand the defect features in strip-pattern-spheres, we further investigated the formation mechanism of the self-assemblies. Critical water content (CWC), an indication for the onset of aggregation, was measured according to the method described in reference 54 From the turbidity (optical density) curves (Fig. 6a), we found that the CWC of PS and PBLG-*b*-PEG are 9.1 vol% and 14.4 vol%, respectively. The PBLG-*b*-PEG/PS mixtures have a CWC of 9.0 vol% which is close to the CWC of PS homopolymers. In mixture system, due to the distinction of CWC, it is reasonable to believe that PS homopolymers firstly self-assemble into aggregates followed by gradual assembly of PBLG-*b*-PEG block copolymers on the surfaces of the pre-formed PS spheres. The formation process of the strip-pattern-spheres in this work is similar to the formation of superhelical structures that have been reported in our previous work, which reveals that PBLG-*b*-PEG block copolymers self-assemble on the pre-aggregated PBLG homopolymer substrates^18^. Indeed, SEM images show that the morphology transforms from plain sphere to strip-pattern-spheres as the added water content increases from 9.0 to 15.0 vol%. At the water content of 9.0 vol% which is almost the CWC of PS solution, smooth spheres with diameter of about 100 nm can be observed (Fig. 6b). Figure 6c shows that larger spheres can be observed at the water content of 10.0 vol%. When water content reaches 14.4 vol%, PBLG-*b*-PEG block copolymers start assembling on the surfaces of PS cores. Increasing the water content to 15.0 vol%, turgid sphere structure with striped patterns is induced (Fig. 6d). After dialysis in water, strip-pattern-spheres with defect features can be clearly observed (Fig. 6e). Upon the formation of striped patterns, +1/2 disclinations are produced due to the constraint of spherical template^35^. Moreover, entropy of polymer chains leads to the disorder in the packing of rigid PBLG rods, which causes the appearance of dislocations in the striped patterns^26^. A scheme of such process is illustrated in Fig. 6f.

To further understand the mechanism of the cooperative self-assembly, some control experiments were designed and carried out. As revealed by literature^55^, phosphotungstic acid (PTA) can react with ester group in PBLG and stain the PBLG contained samples. In a separate experiment, we stained the PBLG-*b*-PEG/PS spheres with PTA. It was found that the strips in the self-assembled structures became clear after positive staining, as compared with the unstained samples (Supporting Information, Figure S6). This further verified the fact that the strips were formed by orderly packed PBLG-*b*-PEG block copolymers. The DLS measurements of the PS and PBLG-*b*-PEG/PS mixture solutions were conducted to confirm the formation mechanism of the strip-pattern-spheres. The polymer concentrations of both solutions were 0.1 g/L, and the initial solvents were THF/DMF mixture solvents (1/1 by volume). Water was slowly dropped into the initial solutions to a content of 10.0 vol%. As shown by Figure S8, the radii and distributions of the two solutions well match with each other, which indicates that the aggregates in the PBLG-*b*-PEG/PS mixture solution are mainly formed by the homo-PS component and the PBLG-*b*-PEG block copolymers still dissolve in the solution (for more details, see Supporting Information, Figure S8). If the water is continuously added to the PBLG-*b*-PEG/PS mixture solution, the PBLG-*b*-PEG block copolymers could start self-assembling into strips on the surface of PS cores. Moreover, we designed a two-step assembling process to prove the formation mechanism of strip-pattern-spheres. In first step, two solutions were prepared. One is PS solution in which the water content is 12.0 vol%. Since the CWC of PS is 9.1 vol%, the PS homopolymers are supposed to aggregate into spheres. This is confirmed by SEM which is shown in Figure S9a. The other is PBLG-*b*-PEG solution with the same water content of 12.0 vol%. It is below the CWC of PBLG-*b*-PEG, thus the block copolymers are in dispersed state. Then the two solutions were mixed and dialysized against water. The SEM image clearly shows that strip-pattern-spheres are obtained (Supporting Information, Figure S9b). Through such a two-step self-assembly experiment, the proposed mechanism, *i.e.*, the PS homopolymers form spheres on which PBLG-*b*-PEG self-assemble into strips, is further confirmed.

We additionally performed a dissipative particle dynamic (DPD) simulation to get deep insight into the mechanism of the self-assembly process (Supporting Information, Figure S2). Initially, all the coarse-grained molecules were randomly set in the simulation box, which can correspond to polymer mixtures in THF/DMF solutions. The simulation results show that, until 250τ, P_20_ homopolymers form into spheres firstly without adsorption of R_4_C_4_ copolymers. At 500τ, as the radii of spheres are as large as 3*r*_c_, R_4_C_4_ diblock copolymers start self-assembling onto the surfaces of spheres. The results of density profiles indicate that the PBLG-*b*-PEG copolymers are almost completely excluded from the PS spheres when a small amount of water is added in the experiment. The simulation proved the formation of stripe-pattern-spheres follows a two-step self-assembly manner.

## Discussion

Based on the combination studies of experiments and DPD simulations, we proposed the arrangement of polymers in the self-assembled strip-pattern-spheres. The hydrophobic rigid PBLG blocks packed orderly to form the inner part of strips on the sphere surface, while PEG blocks stabilized the strip-pattern-spheres in solution. Due to the ordered packing of rigid strip-forming PBLG blocks, the width of the strips remains constant in spite of the variation of experimental conditions, which is one of the distinctive features compared with coil-coil block copolymer strip-forming systems. The period of striped patterns in thin film formed by coil-coil block copolymers changes with experimental conditions^46^.

In addition to striped patterns formed by coil-coil block copolymers in thin film, periodic patterned structures formed by rod-coil block copolymers have been reported in earlier works. For example, Thomas *et al*. reported that rod-coil diblock copolymer poly(3-(triethoxysilyl)propylisocyanate)-*block*-polystyrene (PIC-*b*-PS) can self-assemble into two-dimensional periodic nanostructures^56^,^57^. In their systems, the patterned structure was formed by the microphase separation of the two distinct polymer blocks and the crystallization of the PIC blocks significantly influences the morphology of the periodic structure. Comparing with the periodic patterned structures formed by PIC-*b*-PS, the self-assembled strip-pattern-spheres in our system have different characters. The phase separation of PBLG-*b*-PEG copolymers occurs in the dilute solution of polymers due to the distinct water selectivity of the PBLG and PEG blocks in our system. The ordered packing of PBLG blocks plays an important role in the formation of the strips. Moreover, the striped pattern structures in our system are self-assembled on the surface of spherical PS templates. The constraint of geometric structure results in different defect features on curved surface.

As revealed by the statistical data of defect densities in this work, defects on the spherical surfaces show distinctive features compared with the defects on planar surfaces^22^,^23^,^24^,^25^,^26^,^27^,^28^. When the strips of block copolymers is confined on spherical substrates, requirement of topological constraints should satisfy the Poincaré-Hopf theorem^43^, which means the configurations of strips confined on the spherical surfaces have +1/2 disclinations that cannot be annihilated. The existence of +1/2 disclinations in striped patterns on the spherical surfaces is an important natural geometrical phenomenon. The density of +1/2 disclination is higher than density of −1/2 disclination, and each sphere has four +1/2 disclinations when there is no −1/2 disclination. For the defects in two-dimensional planar striped patterns, two couples of +1/2 and −1/2 disclinations (quadrupoles) can be annihilated, leading to an area of defect-free striped patterns^26^. Therefore, the defect densities of +1/2 and −1/2 disclinations should always be the equal during temperature variation, which unlike the defects on spherical surfaces.

The present work provides a facile self-assembly method to fabricate spherical structures with striped patterns on their surfaces. The striped patterns are self-assembled from the ordered packing of rod-coil block copolymers on the surface of spherical homopolymer templates. Learning from the self-assembling mechanism, we can fabricate striped pattern structures on diverse templates. For example, two-dimensional striped patterns can be obtained by replacing the spherical templates with planar substrates. Therefore, information gained from this work may provide guidance for fabrication of hierarchical supramolecular structures with patterns. Moreover, in the self-assembled strip-pattern-spheres, the inner core template polymer is covered by the pattern-arranged polypeptides. This structure is reminiscent of the Poxviridae viral particle which carries its DNA in envelope with pattern structures on surface. Due to the similarity in morphology, the strip-pattern-spheres obtained in the present system have potential applications in bionics.

In summary, we discovered that the PBLG-*b*-PEG block copolymers and PS homopolymers mixtures could self-assemble into spherical structures with striped patterns in aqueous solution. The hydrophobic PS homopolymers formed the spherical template covered by PBLG-*b*-PEG amphiphilic block copolymers in the surface through attraction between hydrophobic PBLG and PS blocks. In the striped patterns, three kinds of defects, including dislocations, +1/2 disclinations and −1/2 disclinations were observed. The densities of these three types of defects exhibit different dependence on influencing parameters such as temperature and sphere radius. Increasing temperature leads to higher densities of all the three types of defects and less regular patterns. Larger sphere radius results in the decrease of +1/2 disclination density while the density of −1/2 disclinations and dislocations show no obvious change. The present work provides an insight into the formation mechanism of striped pattern structures self-assembled from the polypeptide-based polymer mixtures.

## Methods

### Polymer synthesis

Poly(γ-benzyl-L-glutamate)-*block*-poly(ethylene glycol) (PBLG-*b*-PEG) block copolymers were synthesized by ring-opening polymerization of γ-Benzyl-L-glutamate-N-carboxyanhydride (BLG-NCA) in anhydrous 1,4-dioxane initiated by mPEG-NH_2_ macroinitiator^58^. At the end of the polymerization, the viscous reaction mixture was poured into a large volume of anhydrous ethanol. The precipitated product was filtered and then dried under vacuum. Polystyrene (PS) homopolymer was synthesized by atom transfer radical polymerization (ATRP) with Ethyl 2-bromoisobutyrate (EtBriB) as initiator. After polymerization, copper salts in the mixture were removed through a plugged column of neutral aluminum oxide. The sample was precipitated with cold methanol and then dried under vacuum overnight. Details about the synthesis of polymers are shown in Supporting Information Section 1.1.

### Preparation of assemblies

Polymer assemblies were prepared by adding water to the organic solution of polymer mixtures. PBLG_12100_-*b*-PEG_5000_ block copolymers and PS_19400_ homopolymers were firstly dissolved in mixture solvent of THF/DMF (tetrahydrofuran/*N*,*N′*-dimethylformamide, 1/1, v/v) at a concentration of 0.2 g L^−1^. And then deionized water, a selective solvent for PEG block, was dropped into the initial solution at a rate of *ca.* 1 mL min^−1^ with vigorous stirring. During water addition, the colorless solution gradually took on a blue tint, which indicated the formation of assemblies. After being stabilized for about 30 min, the solution was dialyzed against deionized water for 3 days to remove organic solvents. After dialysis, the obtained hierarchical structures were stabilized. In order to examine the effect of temperature, the entire experiment was performed under constant corresponding temperature (20 °C, 25 °C, 30 °C, 35 °C), including water adding and dialysis process. The polymer solutions, water for micellization and dialysis were stored at corresponding temperature for more than 3 h before use. For investigating the effect of sphere radius, the weight fraction of PBLG-*b*-PEG block copolymers (*f*_PBLG-*b*-PEG_) in the polymer mixtures varied (the initial concentration was fixed at 0.2 g L^−1^).

### Characterization of assemblies

The morphologies were characterized by SEM (S4800, Hitachi, 15 kV), TEM (JEM-2100F, JEOL, 200 kV), cryo-TEM (JEM-2200FS, CEVS, 200 kV, −174 °C), and AFM (XE-100, Park Systems, Non-contact mode). The radii of the aggregates were measured by DLS (CGS-5022F, ALV, laser wavelength 632.8 nm). Detailed experimental information is available in the Supporting Information Section 1.4.

### Simulation methods

The dissipative particle dynamics (DPD) is a mesoscopic simulation method originated by Hoogerbrugge and Koelman^59^, developed by Robert and Patrick^60^. In this method several neighboring molecules are grouped into a single particle. The Newton’s equations of motion are applied for calculating the trajectories of beads in the system. Using a modified velocity-Verlet algorithm, the positions and velocities of all the beads are propagated. According to DPD method the force **F**_*i*_ applying on a coarse-grained DPD bead *i* is a sum of conservative force, dissipative force, and random force (details are shown in Supporting Information Section 2). For diblock copolymers, an additional harmonic spring potential 
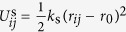
 is applied to each pair of two bonded beads *i* and *j*, where *r*_0_ and *k*_s_ denote the equilibrium bond distance and bond spring constant, respectively. The rigidity of rod blocks is realized by cosine harmonic function, which keeps the angle formed by each three beads to a constant value: the chain stiffness potential 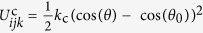 is performed on three neighbored beads *i, j*, and *k* in rod blocks, in which *θ*_0_ and *k*_c_ correspond to the equilibrium angle and angle spring constant, respectively. The simulation boxes, with periodical boundary condition, have varied sizes according to the radii of spherical substrates. The sphere is modeled to a hollow spherical substrate formed by beads. The block copolymer beads and water beads were filled outside the spherical substrate. More simulation information is available in the Supporting Information Section 2.

## Additional Information

**How to cite this article**: Zhu, X. *et al*. Strip-Pattern-Spheres Self-Assembled from Polypeptide-Based Polymer Mixtures: Structure and Defect Features. *Sci. Rep.* **6**, 29796; doi: 10.1038/srep29796 (2016).

## Supplementary Material

Supplementary Information
