# Supplementary material for: Strip-Pattern-Spheres Self-Assembled from Polypeptide-Based Polymer Mixtures: Structure and Defect Features
Source: Sci Rep. 2016 Jul 15;6:29796. doi: 10.1038/srep29796 (PMC4945953; doi:10.1038/srep29796)
Supplement: Supplementary Information [file srep29796-s1.doc]

**Supporting Information**

Strip-Pattern-Spheres Self-Assembled from Polypeptide-Based Polymer Mixtures: Structure and Defect Features

Xingyu Zhu, Zhou Guan, Jiaping Lin,* Chunhua Cai*

Shanghai Key Laboratory of Advanced Polymeric Materials, State Key Laboratory of Bioreactor Engineering, Key Laboratory for Ultrafine Materials of Ministry of Education, School of Materials Science and Engineering, East China University of Science and Technology, Shanghai 200237, China *Corresponding Author. Tel: +86-21-6425-3370. E-mail: jlin@ecust.edu.cn; caichunhua@ecust.edu.cn.

**1. Experimental Section**

**1.1 Polymer Synthesis**

**Materials***.* α-Methoxy-ω-amino poly(ethylene glycol) (mPEG-NH_2_, *M*_n_ = 5000) was purchased from Sigma-Aldrich Co., Inc. mPEG-NH_2_ macroinitiator was dried by dissolving in toluene and then toluene was removed in high vacuum before use. Analytical grade of hexane and 1,4-dioxane were refluxed with sodium and distilled immediately before use. Acetic ether was refluxed and distilled with CaH_2_. Styrene was distilled over CaH_2_ under reduced pressure to remove inhibitor. CuBr was purified by stirring in acetone overnight, filtered and washed with acetone for three times then dried under vacuum at room temperature. Ethyl 2-bromoisobutyrate (EtBriB) and 1,1,4,7,7-pentamethyldiethylenetriamine (PMDETA) were purchased from Adamas-beta Co., Inc. and used as received. The dialysis bag (Membra-cel, 3500 molecular weight cutoff) was provided by Serva Electrophoresis GmbH. All the other reagents were purchased from Adamas-beta Co., Inc. and used as received. Deionized water was made from a Millipore Super-Q Plus Water System to a level of 18.2 MΩ cm resistance.

**Synthesis of PBLG-*b*-PEG block copolymer.** γ-Benzyl-L-glutamate-N-carboxyanhydride (BLG-NCA) was synthesized according to the literature procedure.^S1,S2^ Poly(γ-benzyl-L-glutamate)-*block*-poly(ethylene glycol) (PBLG-*b*-PEG) block copolymers were synthesized by ring-opening polymerization of BLG-NCA in anhydrous 1,4-dioxane initiated by mPEG-NH_2_ macroinitiator according to our previous work.^S3,S4^ The monomer concentration is *ca.* 3.0 wt%. The reaction was performed in flame-dried reaction bottle under a dry nitrogen atmosphere for 3 days at 15 °C. At the end of the polymerization, the viscous reaction mixture was poured into a large volume of anhydrous ethanol. The precipitated product was filtered and then dried under vacuum.

**Synthesis of PS homopolymer.** Polystyrene homopolymer was synthesized by atom transfer radical polymerization (ATRP) with EtBriB as initiator.^S5^ 56 mg (0.3 mmol) EtBriB, 20.6 g (200 mmol) distilled styrene, 43 mg (0.3 mmol) CuBr, and 103 mg (0.6 mmol) PMDETA were added into a Schlenk tube. The mixture was purged with dry nitrogen and subjected to three freeze-pump-thaw cycles to remove any dissolved oxygen. The tube was then sealed with nitrogen and immersed in a thermostatic oil bath at 70 ^o^C. After 24 h, the mixture was diluted with tetrahydrofuran and stirred under air to stop the reaction. Then copper salts in the mixture were removed through a plugged column of neutral aluminum oxide. After concentrating the solvent by reduced pressure distillation, the homopolymer was precipitated in a large volume of cold methanol. The sample was dried under vacuum overnight.

**1.2 Polymer characterizations**

The molecule weights of PBLG-*b*-PEG block copolymer and PS homopolymer were estimated using ^1^H NMR measurement (Avance 550, Nuclear, CDCl_3_ as solvent) and gel permeation chromatography (PL-GPC, Varian, PS as standard). The molecular weight of PEG segment is known (M_n_ = 5000), and the degree of polymerization of PBLG segment can be estimated by ^1^H NMR and GPC measurements.^S3-S6^ The structure information of PBLG-*b*-PEG block copolymer and PS homopolymer is provided in **Table S1**.

**Table S1. Polymer characterizations**

| Molecular structure | Sample | *M*_n_^a^ | DP^a^ | PDI^b^ |
| --- | --- | --- | --- | --- |
|  | PBLG-*b*-PEG-1 | PEG: 5000  PBLG:12100 | PEG: 112  PBLG: 55 | 1.13 |
|  | PBLG-*b*-PEG-2 | PEG: 5000  PBLG:31000 | PEG: 112  PBLG: 141 | 1.19 |
|  | PBLG-*b*-PEG-3 | PEG: 5000  PBLG:56000 | PEG: 112  PBLG: 255 | 1.22 |
|  | PS-*b*-PEG | PEG: 5000  PS:15000 | PEG: 112  PS: 144 | 1.04 |
|  | PS | 19400 | 186 | 1.05 |

^a^The number-average molecular weight (*M*_n_) of PS homopolymer was obtained from the gel permeation chromatography (GPC) analysis with THF as elution solvent; for PBLG-*b*-PEG and PS-*b*-PEG block copolymer, the *M*_n_ value and the degree of polymerization (DP) of PEG segment was known, and the *M*_n_ and DP of PBLG block were derived according to ^1^H NMR spectra.

^b^The PDI values of the polymers were obtained from GPC testing.

**1.3 Sample preparation**

Firstly, PBLG-*b*-PEG block copolymers and PS homopolymers were dissolved in tetrahydrofuran (THF)/*N*,*N’*-dimethylformamide (DMF) (1/1, v/v) mixture solvents at a concentration of 0.2 g L^-1^ by stirring at corresponding temperature for 1 day, respectively. Then block copolymer and homopolymer solutions were mixed with designed volume ratio (for example, 1:1, that is, 2 mL PBLG-*b*-PEG block copolymer solution and 2 mL PS homopolymer solution were mixed) for system applied to explore the effect of self-assembling temperature. To prepare self-assemblies, typically, 1.5 mL of deionized water was added to 4 mL of initial solution with vigorous stirring. After the addition of water, the colorless solution became tint blue, which indicated the formation of self-assembled structures.^S7^ The solution was dialyzed against deionized water for 3 days to ensure that all the organic solvents were removed.

To study the effect of self-assembling temperature, the experiments were conducted at various temperatures of 15 ^o^C, 20 ^o^C, 25 ^o^C, 30 ^o^C, and 35 ^o^C, the polymer concentration was fixed to 0.2 g L^-1^, and the PBLG-*b*-PEG weight fraction (*f*_PBLG-_*_b_*_-PEG_) was 0.5. Polymer solutions and water used for self-assemble procedures were stored at corresponding temperature for at least 12 h. Then all of the experiments, including the adding water and dialysis process, were performed at the constant temperature. The storage temperature has negligible effect on structures of the self-assembled aggregates. After storing the solution at different temperatures for more than 3 months, there was no morphology transformation observed.

For studies of radius effect, the experiments were conducted with various PBLG-*b*-PEG weight fractions (*f*_PBLG-_*_b_*_-PEG_) of 0.8, 0.6, 0.4, and 0.2, the total polymer concentration was fixed to 0.2 g L^-1^, and the self-assembly and dialysis temperature was 20 ^o^C.

**1.4 Aggregate structure characterizations**

**Scanning Electron Microscopy (SEM).** The morphologies of aggregates were observed by Field Emission SEM (S4800, HITACHI) operated at an accelerating voltage of 15 kV. The samples were prepared by placing drops of solution on a copper grid coated with carbon film and then dried at room temperature. Before observations, the samples were sputtered by gold.

**Transmission Electron Microscopy (TEM).** The morphologies of aggregates were examined by Field Emission TEM (JEM-2100F, JEOL) operated at an accelerating voltage of 200 kV. Drops of solution were placed on a copper grid coated with carbon film and then dried at room temperature.

**Atom Force Microscopy (AFM).** AFM measurements were performed with XE-100 (Park Systems) by using the non-contact mode at room temperature in air. The samples were prepared by placing drops of solution on a silicon wafer surface and allowed to dry in air.

**Cryo-TEM.** Cryo-TEM samples were prepared in a controlled environment vitrification system (CEVS). One drop of solution was placed on a copper grid coated with carbon film. The excess solution was blotted with a piece of filter paper, and then quickly dipped into liquid ethane, which was cooled by liquid nitrogen. The vitrified samples were then stored in liquid nitrogen until they were transferred to cryogenic sample holder (Gatan 626) and examined with JEM-2200FS TEM at an accelerating voltage of 200 kV at temperature about -174 ^o^C.

**Dynamic Light Scattering (DLS).** DLS was measured by an LLS spectrometer (ALV/CGS-5022F) equipped with an ALV-High QE APD detector and an ALV-5000 digital correlator using a He−Ne laser (the wavelength *λ* = 632.8 nm) as the light source. All the samples were measured without filteration, and the measurements were carried out at 20 °C. The scattering angle is 90°. From DLS testing, hydrodynamic radius (*R*_h_) can be obtained, which indicates the radius of a sphere with the same translational diffusion coefficient and the same condition.^S8^

**Turbidity measurements (optical density).** Turbidity measurements were performed to determine the critical water concentration (CWC) for the aggregate formation.^S9^ The polymers were first dissolved into THF/DMF (1/1, v/v) mixture solvents at a concentration of 0.2 g L^-1^, which is the same condition for the self-assembling process. Deionized water was then added drop by drop (10 *μ*L per drop to 2 mL of polymer solution) under magnetic stirring. The optical density (turbidity) was measured at a wavelength of 690 nm using a quartz cell (path length: 1 cm) with a UV-Vis spectrophotometer (UV-2550 SHIMADZU).

**2. Simulation Section**

**2.1 Dissipative particle dynamic (DPD) methods**

The dissipative particle dynamics (DPD) is a mesoscopic simulation method originated by Hoogerbrugge and Koelman,^S10,S11^ developed by Robert and Patrick.^S12^ In this method several neighboring molecules are grouped into a single particle. The Newton’s equations of motion are applied for the position-time relationship of every bead in the system. Using a modified velocity-Verlet algorithm, all beads’ positions and velocities are integrated.

According to DPD method the force **F***_i_* applying on a coarse-grained DPD bead *i* is a sum of conservative force, dissipative force, and random force, represented as the following equation:

$\mathbf{F}_{i}=\sum_{i\neq j} \left(\mathbf{F}_{ij}^{\text{C}}+\mathbf{F}_{ij}^{\text{D}}+\mathbf{F}_{ij}^{\text{R}}\right)$ (1)

The three kinds of forces on the right hand of the above equation take the forms as follows:

$\mathbf{F}_{ij}^{\text{C}}=\left\{\begin{matrix} a_{ij}\left(1-r_{ij}/r_{\text{c}}\right)\hat{\mathbf{r}}_{ij} & \text{ }r_{ij}<r_{\text{c}} \\ 0 & \text{ }r_{ij}\geq r_{\text{c}} \end{matrix}\right.$ (2)

$\mathbf{F}_{ij}^{\text{D}}=\left\{\begin{matrix} -\gamma\left(1-r_{ij}/r_{\text{c}}\right)^{2}\left(\hat{\mathbf{r}}_{ij}\cdot\mathbf{v}_{ij}\right)\hat{\mathbf{r}}_{ij} & \text{ }r_{ij}<r_{\text{c}} \\ 0 & \text{ }r_{ij}\geq r_{\text{c}} \end{matrix}\right.$ (3)

$\mathbf{F}_{ij}^{\text{R}}=\left\{\begin{matrix} \left(2\gamma k_{\text{B}}T\right)^{1/2}\left(1-r_{ij}/r_{\text{c}}\right)\theta_{ij}\left(dt\right)^{-1/2}\hat{\mathbf{r}}_{ij} & r_{ij}<r_{\text{c}} \\ 0 & r_{ij}\geq r_{\text{c}} \end{matrix}\right.$ (4)

*a_ij_* is the repulsive parameter between two arbitrary beads *i* and *j*, and $a_{ij}=75k_{\text{B}}T/\rho+3.27\chi_{ij}$, where *k*_B_, *T*, and *χ_ij_* denotes to Boltzmann constant, tempsrature, and Flory-Huggins parameter, respectively. *r_ij_* is the distance between these two beads *r_ij_* = **|r***_i_*-**r***_j_***|**, **r***_ij_* is the unit vector **r***_ij_* = (**r***_i_*-**r***_j_*)/*r_ij_*. *r*_c_ is the cutoff distance. *γ* is the strength of the dissipation between bead *i*, *j*, and **v***_ij_* = **v***_i_*-**v***_j_*. *θ_ij_* is a random fluctuating variable with Gaussian statistics and has zero mean and unit deviation.

For diblock copolymers, an additional harmonic spring potential $U_{ij}^{\text{s}}=\frac{1}{2}k_{\text{s}}\left(r_{ij}-r_{0}\right)^{2}$ is applied to each pair of two bonded beads *i* and *j*. In this work, the equilibrium bond distance *r*_0_ and *k*_s_ are set to be 0.7 *r*_c_ and 100 *k*_B_*T/r*_c_^-2^, respectively. The rigidity of rod blocks is realized by cosine harmonic function, which keeps the angle formed by each three beads to a constant value: the chain stiffness potential $U_{ijk}^{\text{c}}=\frac{1}{2}k_{\text{c}}\left(\cos\left(\theta\right)-\cos\left(\theta_{0}\right)\right)^{2}$ is performed on three neighbored beads *i*, *j* and *k* in rod blocks. The equilibrium value of the angle *θ*_0_ and the value of *k*_c_ are set as 180° and 100 *k*_B_*T*, respectively. Figure S1a shows the schematic illustration of diblock copolymers model we constructed.

For simplicity, the sphere is modeled to a hollow spherical substrate formed by beads (Figure S1b). To guarantee the uniform arrangement of beads constructing the substrate, we adopted the maximal volume arrangements of spherical codes proposed by Neil et al.^S13^ The average distance between neighbor beads was set close enough to avoid the copolymer and solvents permeating into the ball. The simulation boxes, with periodical boundary condition, had varied sizes according to the radii of spherical substrates. The distance between the spherical substrate and box faces was set to 10 *r*_c_. The block copolymer beads and water beads with density *ρ *= 3 were filled outside the spherical substrate.

Applying velocities with a Gaussian distribution to all the beads except those constructing the spherical substrate, the DPD simulation method was then run for 20000*τ *in order to offer enough time for the systems reaching stable configurations. Under each parameter condition, the simulation was performed 10 times with different random number seeds, and the final quantitative results were averaged from all these 10 times simulations. The time step was set to *dt* = 0.01*τ*, in which$\tau=\sqrt{mr_{\text{c}}^{2}/\left(k_{\text{B}}T\right)}$. The massive of all beads, cutoff distance and temperature were all set unit (*m* = 1.0, *r*_c_ = 1.0 and *k*_B_*T* = 1.0), and the value of *τ* was also assigned as unit.

In initial state the rod-coil diblock copolymers were uniformly arranged on the spherical surfaces along radial directions with rod and coil blocks forming the inner and outer layer, respectively. The initial structures are constructed for the purpose of minimizing the free energy of the systems at the beginning of the simulations, so that the polymers would self-assemble into stable morphologies in short time. After the simulations were performed for 20000 *τ*, the diblock copolymers form stripped patterns on the spherical substrates, as shown in Figure S1c. Figure S1d illustrates the Miller cylindrical projections of the finial structures,^S14^ corresponding to Figure S1c.

**
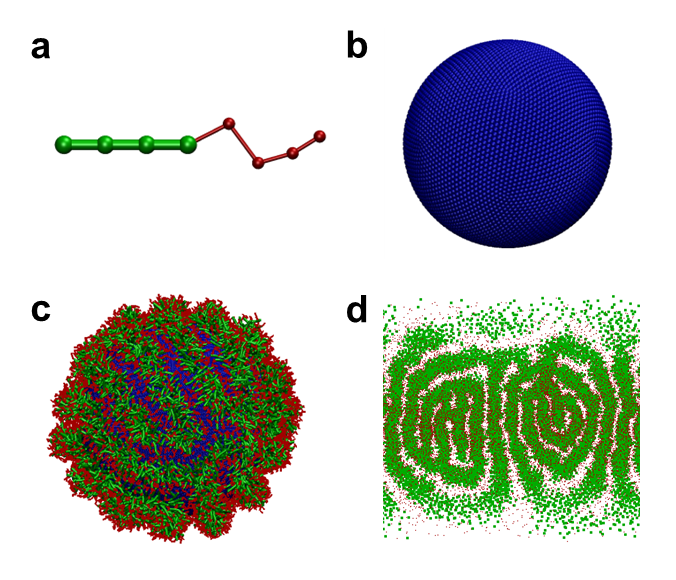
**

**Figure S1. Polymer models and strip-pattern-spheres in simulation.** (a) DPD model of amphiphilic rod-coil block copolymers (green beads stand for rigid hydrophobic rods, red beads stand for coiled hydrophilic block); (b) model of fixed spherical template (blue); (c) typical simulated self-assembled structure; (d) 2D projection image of strip-pattern-sphere in panel (c).

**2.2 Important parameters in simulation methods**

In simulation, we set important parameters based on experimental results to make the simulation system and experimental system close. The followings are the discussion and explanation on the choice of some important parameters.

1. **Bead number**. Keeping the total number of beads in the simulation box constant with density *ρ *= 3, the amount of block copolymer beads may be varied with the change of sphere radius. For simplicity, this value has been simplified to the surface density of copolymers (*N*/*s*), where *N* denotes the number of diblock copolymer chains and *s* denotes the surface area of spherical substrate. The surface density of polymer (*N*/*s*) is set to 1.4 *r*_c_^-2^ in the present work.
2. **Rigidity of molecules**. The PEG is flexible, thus only a bond potential was applied for the coil block in rod-coil copolymers. Since the PBLG homopolymers with α-helix conformation can be considered as rigid segments, in the simulations a bond-angle potential was employed to guarantee the rigidity of rod block by setting a large value of *k*_c_ = 100 *k*_B_*T*.^S15^
3. **Interaction parameter**. To facilitate a comparison with available experiments (PBLG-*b*-PEG/PS binary system in solvents), we set the interaction parameters *a*_ij_ in the DPD simulation close to those in experiment systems. For the beads of the same species, repulsive parameters were set as 25. The repulsive parameters between different types of beads were: *a*_RC_ = 80, *a*_RS_ = 80, *a*_RP_ = 25, *a*_CS_ = 30, *a*_CP_ = 25, and *a*_PS_ = 120. R, C, S, and P denote rod blocks (PBLG), coil blocks (PEG), solvents, and the beads constructing the spherical substrate (PS), respectively. These parameters were set corresponding to the hydrophobicity of PBLG blocks and PS homopolymers and the hydrophilicity of PEG blocks. The value of *a*_PS_ was larger than the others to make sure the diblock copolymers attaching to the spherical substrate without dissolving in the solvents.

Through such choices of parameters, we found the simulation results are able to capture the characteristic of the experimental systems. For example, typical features such as defect evolution tendency can be predicated by simulations.

**2.3 Self-assembly process of strip-pattern-spheres**

We performed a DPD simulation to get deep insight into the mechanism of the self-assembly process. The simulation box, with size 40×40×40 *r*_c_^3^ and density *ρ* = 3, was filled with 1000 P_20_ homopolymers, 1000 R_4_C_4_ block copolymers, and 164000 S solvent beads. The P_20_ homopolymers, R_4_ and C_4_ segments, and S corresponds to PS homopolymers, PBLG and PEG blocks, and water in the experiments, respectively. All the parameters were set corresponding to the experimental ones, which are the same with those in the DPD simulations of present work.

Initially, all the coarse-grained molecules were randomly set in the simulation box (Figure S2a), which can correspond to polymer mixtures in THF/DMF solutions. An acting of DPD simulation denotes the addition of water to the solution. The simulation results show that, until 250*τ*, P_20_ homopolymers form into spheres firstly without adsorption of R_4_C_4_ copolymers (Figure S2b), which indicates a prior phase separation of PS homopolymers in experiment. Then the sizes of homopolymer spheres increase. At 500*τ*, as the radii of spheres are as large as 3*r*_c_, R_4_C_4_ diblock copolymers start self-assembling onto the surfaces of spheres (Figure S2c). This process corresponds to the adsorption of PBLG-*b*-PEG copolymers on the spherical substrates of PS aggregates.

Then we examined the density profiles of PS, PBLG, and PEG blocks in an aggregate at 250*τ* and 500*τ*, the results are shown in Figure S2d and e. It can be seen that, in the first self-assembly step (250*τ*), P_20_ homopolymer spheres are formed, and R_4_C_4_ copolymers remain dissolved in the solvents. The result indicates that the PBLG-*b*-PEG copolymers are almost completely excluded from the PS spheres when a small amount of water is added in the experiment. This is because of the strong immiscibility between PS and PBLG-*b*-PEG, and the different water-solubility of PS and PBLG-*b*-PEG (CWC of PS is much lower than that of PBLG-*b*-PEG). In the second self-assembly step (500*τ*), as shown in Figure S2e, the densities of copolymers are increased near the aggregate shell, which means PS core and PBLG-*b*-PEG shell are formed in the spherical aggregates. The simulation proved the formation of stripe-pattern-spheres follows a two-step self-assembly manner.


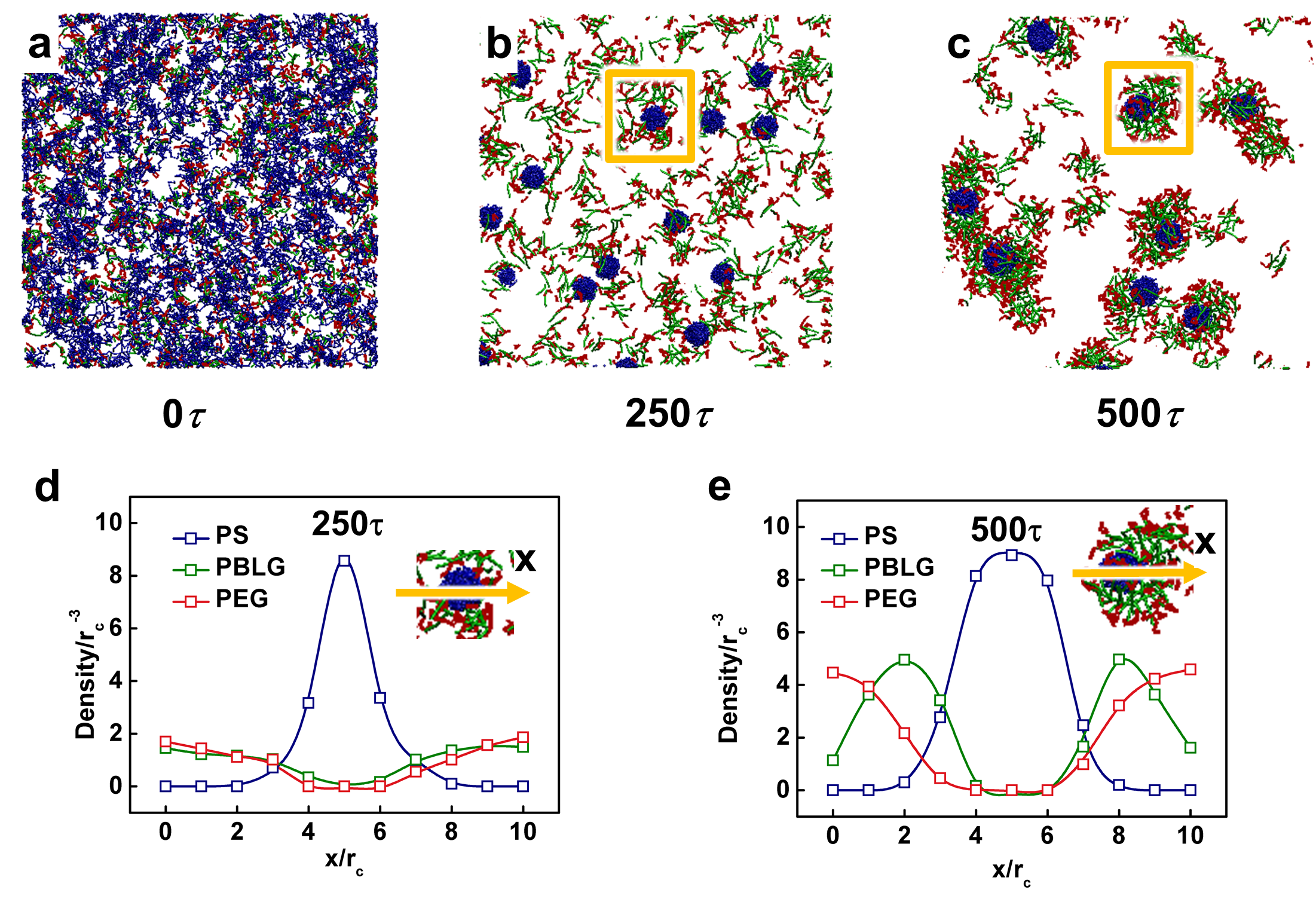


**Figure S2. Self-assembly process strip-pattern-spheres.** (a) configuration of initial system; (b) homopolymers firstly aggregate to spheres; (c) block copolymers self-assemble onto the surfaces of homopolymer spheres. (d, e) Densities of PS, PBLG, and PEG blocks marked by orange squares in b and c. Blue, green, and red denote PS, PBLG, and PEG, respectively. Solvents are not shown for clarity.

**3. Experimental Observations**

**3.1 Defect classification**

There is a well-known theory about defects for materials such as solid crystals, nematic liquid crystals, and other types of substance.^S16-S19^ The method, according to such theory, is applied to classify defects in this self-assembled system. Figure S3 displays schematic illustrations of classical topological defects: (a) dislocation; (b) +1/2 disclination; (c) -1/2 disclination. From the SEM images of aggregates self-assembled from mixture of PBLG-*b*-PEG/PS, these three kinds of defects can be identified. Simulation revealed similar defect patterns, which further verified the result obtained from experiments.


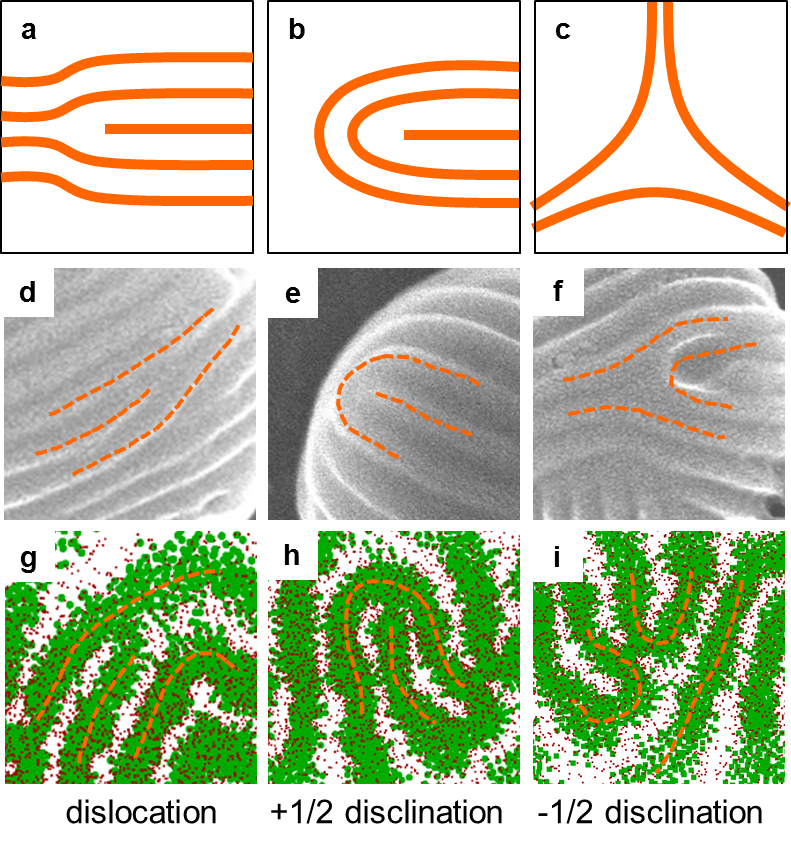


**Figure S3. Typical topological defect configurations.** (a) (d) (g) dislocation; (b) (e) (h) +1/2 disclination; (c) (f) (i) -1/2 disclination. (a)-(c) are schematic illustrations. (d)-(f) are SEM images from experiments. (g)-(i) are 2D projection of striped patterns from simulations.

**3.2 Statistics method**

Every data in Figure 3 was collected from about 50 spheres and the error bars represent standard deviation of all the corresponding samples. To confirm the statistical methods applied in characterization of defects is reliable, we plotted the dependence of average defect number on the number of sphere that was taken into statistics (Figure S4). The striped pattern structure was self-assembled at 20 ^o^C with PBLG-*b*-PEG weight fraction of 0.5. Every sphere was chosen randomly and then the defect feature on its surface was examined. Figure S4 shows a convergence tendency of all the three kinds of defects, which means the statistic data is reliable and stable with more than 30 spheres taken into considerations.^S20^ In simulation part, every data point was calculated for 10 times with different random number seed while the other parameters remain the same. Figure 5 in the main text presents the defect densities and their errors in simulations.


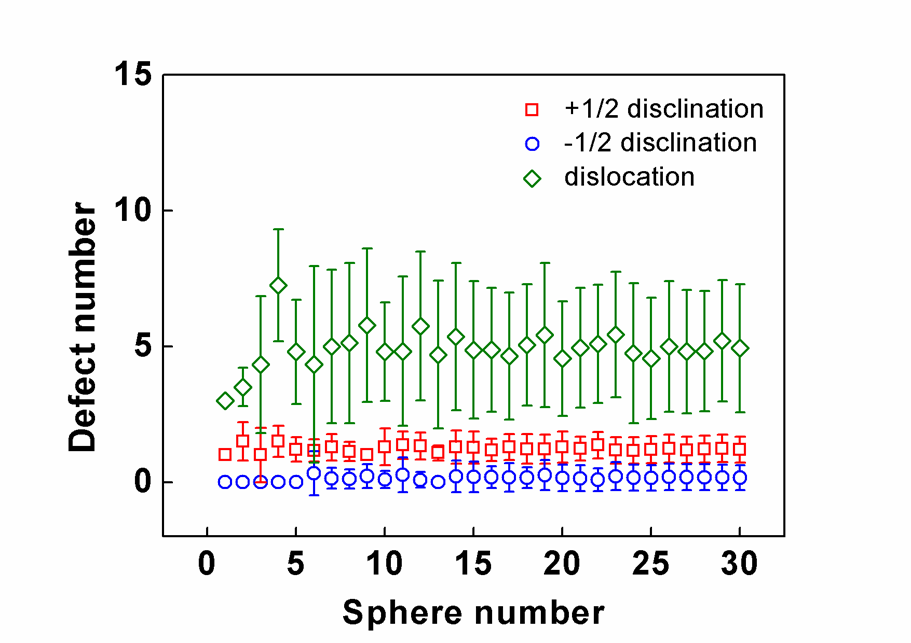


**Figure S4. The relationship between average defect number and sphere number.**

**3.3 Statistic results of strip width and pitch size**

As revealed by our work, parameters such as self-assembling temperature and sphere radius had effects on defect densities of the striped patterns. In addition, the effects of temperature and sphere radius on pitch size and strip width in the self-assembled structure were investigated. Figure S5a shows the illustration of pitch size and strip width. Although the temperature increases, the width of strips and pitch of the patterns remain almost the same according to statistic results (Figure S5b). In order to examine the effect of sphere radius on the defect density in experiments, we manipulated the radius of self-assembled aggregates by changing the weight fraction of PBLG-*b*-PEG (*f*_PBLG-_*_b_*_-PEG_) in the PBLG-*b*-PEG/PS mixtures. As shown in Figure S5c, although the mixture ratio of block copolymer to homopolymer varies, the width of strips and pitch of the patterns remain the same. In Figure S5b and S5c, every data was measured from more than 10 SEM images.

As revealed by simulations, the hydrophobic rods lie closely on the surface of the template sphere. The packing of B rods is in a way that the free ends are in center of the strips and the ends connected to A segments are close to the shell. And the rods are ordered perpendicular to the strip axis. As shown in the SEM and AFM images, the striped patterns have almost uniform pitch size (*ca.* 69nm). For PBLG in α-helix conformation, every helix contains 3.6 amino acid units, and the screw-pitch is 0.54 nm. Therefore, in our experiments, the length of rigid PBLG blocks with 55 units is estimated to be 8.25 nm. For flexible PEG segments, one PEG unit occupies 0.35 nm.^S5^ Therefore, the fully stretched PEG block with 112 units can theoretically reaches 39.2 nm. It is known from simulations that the pitch size (*ca.* 69 nm) equals twice the length of block copolymers. Under such circumstance, each block copolymer occupies 34.5 nm, which means that the PEG chains are stretched to 26.25 nm (stretching degree 67%). In aqueous solution, the PEG segments are highly stretched to cover all area of the sphere. In this case, such a stretching degree is reasonable.

In comparison to the results obtained by experiments, the average pitch size and strip width were taken into statistic by a similar method from simulation results. Figure S5d shows the illustration of pitch size and strip width in simulation results. Figure S5e and S5f show that pitch size and strip width remain constant with the variation of *a*_RS_ and *R*, respectively, which further verifies the results from experiments.

**
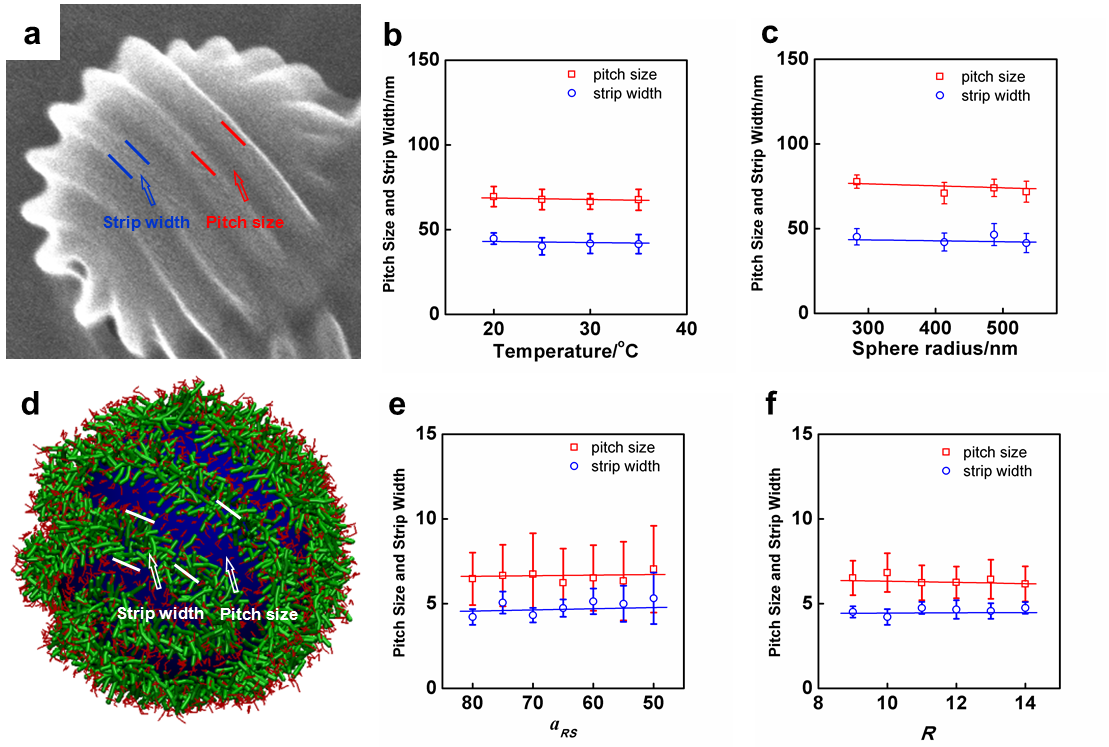
**

**Figure S5. Strip width and pitch size in experiments and simulations.** (a) Schematic illustration of pitch size (red) and strip width (blue) from SEM image; (b) The dependence of average pitch size and strip width on temperature; (c) The dependence of average pitch size and strip width on sphere radius; (d) Schematic illustration of pitch size and strip width from simulations; (e) The dependence of average pitch size and strip width on *a*_RS_; (f) The dependence of average pitch size and strip width on *R*.

**3.4 Comparison of TEM images for unstained and stained samples**

Figure S6a shows the TEM image of self-assembled striped pattern on spherical structures without staining. The sample shown in Figure S6b was stained by phosphotungstic acid (PTA). One drop of 0.1 wt% PTA water solution was dropped onto the sample, and then the sample was washed by pure water after drying. According to earlier literature,^S21^ PTA reacts with ester group as positive stain, which means PBLG component can be stained by PTA. Comparing with Figure S6a, the strips in Figure S6b were obviously stained. Through the TEM staining results, the fact that the stripped patterns are formed by PBLG-*b*-PEG block copolymers is further verified.

**
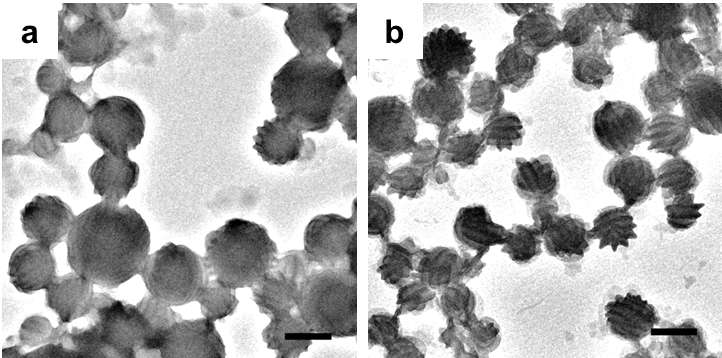
**

**Figure S6. Comparison of unstained and stained samples under TEM.** TEM images of self-assembled striped pattern on spherical structures: (a) without staining; (b) stained by PTA. Scale bars: 400 nm.

**3.5 Stability studies of self-assembled striped patterns on spherical structures**

The striped pattern structure on the aggregates is stable after dialysis process in water. Figure S7 shows the TEM and SEM images of self-assembled striped pattern on spherical structures after being aged for more than 3 months. Striped patterns were still clear and the number of defects hardly changed. Stabilized by hydrophilic PEG shell, the aggregates neither precipitated nor dissociated after being fabricated for 3 months.

**
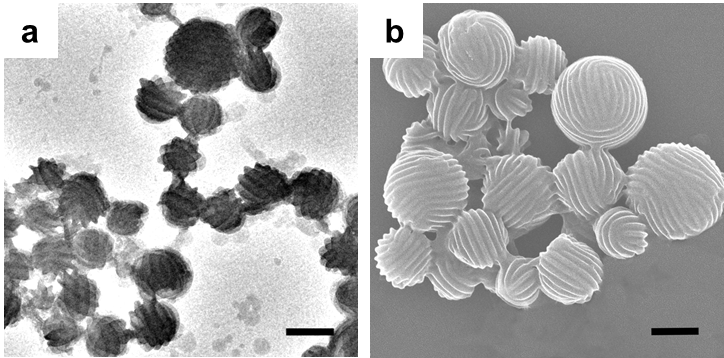
**

**Figure S7. Stability studies of self-assembled striped patterns on spherical structures.** (a) TEM image and (b) SEM image of self-assembled striped pattern on spherical structures after aged for more than 3 months. TEM sample was stained by PTA. Scale bars: 400 nm.

**3.6 DLS measurements of the PS and PBLG-*b*-PEG/PS mixture solutions**

The DLS measurements of the PS and PBLG-*b*-PEG/PS mixture solutions were further conducted to confirm the formation mechanism of the strip-pattern-spheres. The polymer concentrations of both solutions were 0.1 g/L, and the solvents were THF/DMF mixture solvents (1/1 by volume). The weight fraction of block copolymers in the PBLG-*b*-PEG/PS mixture was 0.5. Water was slowly dropped into the solutions to a content of 10.0 vol%. As shown by Figure S8, the hydrodynamic radius (*R*_h_) of sphere aggregates formed from PS solution is *ca.* 166 nm and its distribution index is 0.203. For the aggregates formed from PBLG-*b*-PEG/PS mixture solution, the *R*_h_ is *ca.* 168 nm and the distribution index is 0.221. The radii and distributions of the aggregates in the two solutions well match with each other, which indicates that the aggregates in the PBLG-*b*-PEG/PS mixture solution are mainly formed by the homo-PS component and the PBLG-*b*-PEG block copolymers still dissolve in the solution. Therefore, we think the formation of the strip-pattern-spheres in our system is mainly determined by the mechanism mentioned in the main text.

**
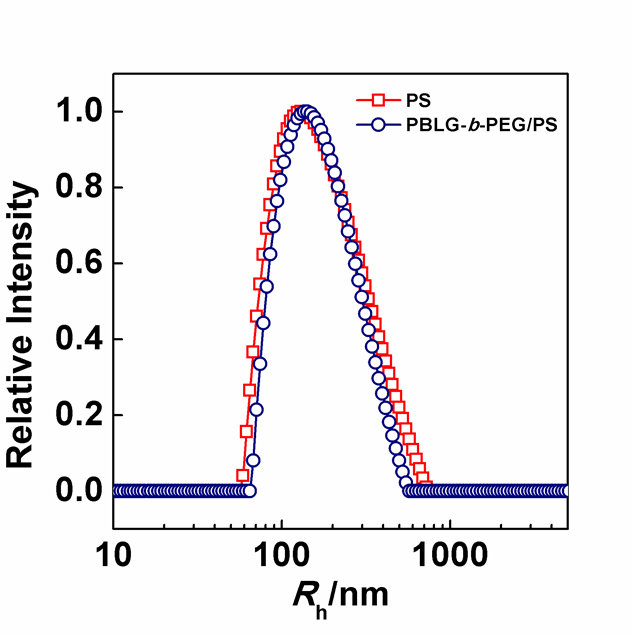
**

**Figure S8. DLS measurements of the PS (red line) and PBLG-*b*-PEG/PS mixture (blue line) solutions.** The solvents were THF/DMF mixture solvent, and the water contents were 10.0 vol%. The scattering angle was 90º.

**3.7 Two-step assembling process of striped pattern structures**

To further verify the formation mechanism of striped pattern on spherical structures, a two-step assembling process was designed and conducted.

First step: water was dropwise added to the solution of PS homopolymers (the initial solvent is 2mL THF/DMF = 1/1 in volume, and the polymer concentration is 0.2 g L^-1^) until the adding water content reached 12.0 vol%. The PS solution with adding water content of 12.0 vol% is denoted by Solution A. Because the CWC of PS solution is 9.1 vol%, the PS homopolymers in Solution A assembled into aggregates. Figure S9a shows the sphere structure of PS aggregates in Solution A. The sample was dried in vacuum immediately after freezing a drop of Solution A on silicon substrate by liquid nitrogen. At the same time, a solution of PBLG-*b*-PEG block copolymers with the same condition of Solution A was prepared (the initial solvent is 2 mL THF/DMF = 1/1 in volume, the polymer concentration is 0.2 g L^-1^, and the water content is 12.0 vol%), which is denoted by Solution B. Since the CWC of PBLG-*b*-PEG solution is 14.4 vol%, the PBLG-*b*-PEG block copolymers are still dissolved.

Second step: equal volume of Solution A and Solution B were mixed and stabilized for 20min, and then the mixed solution was dialyzed against deionized water for 3 days to ensure that all the organic solvents were removed. Figure S8b shows that there are striped patterns on the spherical surfaces obtained by the two-step process. Striped patterns can be observed clearly from the inset of Figure S9b.

In dynamic study of formation process of striped pattern structures, it is believed that PS homopolymers firstly self-assemble into aggregates followed by gradual assembly of PBLG-*b*-PEG block copolymers on the surface of the pre-formed PS spheres. Through the two-step self-assembly of striped pattern on spherical structure, the formation process of the aggregates is further confirmed.


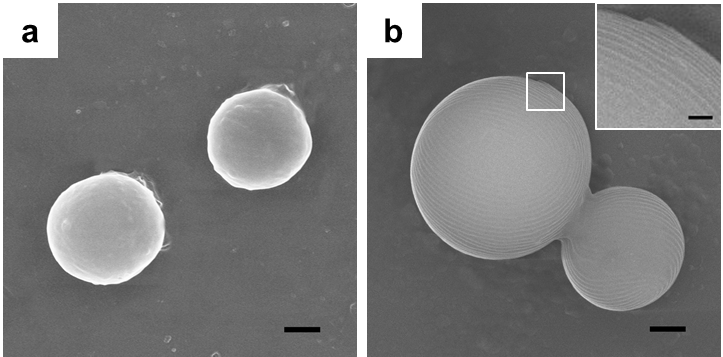


**Figure S9. Two-step assembly of striped pattern structures.** (a) SEM images of spheres assembled from PS homopolymers. The initial solvent is THF/DMF = 1/1 in volume, and the volume ratio of added water to organic solvent is 12.0 vol%. The sample was directly applied to observation, no dialysis process was conducted. (b) SEM images of striped pattern on spherical structure obtained by the two-step process. The inset of (b) shows the detail view of morphology in the white square. Scale bars: 400 nm for (a) and (b), 100 nm for inset of (b).

**3.8 Effects of molecular weight of PBLG blocks and rigidity of hydrophobic segments on the aggregate structures**

In order to prepare strip-pattern-spheres, the molecular weight of PBLG in block copolymers should be moderate. For example, the block copolymer PBLG_12100_-*b*-PEG_5000_ is appropriate for fabricating strip-pattern-spheres. When the PBLG block is longer, it is unlikely to form the same aggregates. The aggregates self-assembled from PBLG_31000_-*b*-PEG_5000_/PS_19400_ are shown in Figure S10a. Some block copolymers aggregate into small spheres instead of assembling on the surface of PS. As shown by Figure S10b, the aggregates formed by PBLG_56000_-*b*-PEG_5000_/PS_19400_ with even longer PBLG blocks are irregular, and the strip-pattern-spheres can hardly be obtained. In addition, many aggregates formed by PBLG-*b*-PEG block copolymers are shown. According to the formation mechanism of strip-pattern-spheres proposed in the main text, the difference of CWC of homopolymers and block copolymers plays determined role in the formation of the strip-pattern-spheres. When the molecular weight of PBLG in block copolymers increases, the weight fraction of hydrophobic blocks becomes higher, leading to relatively lower CWC of the block copolymers. In such a case, the CWC of homopolymers and block copolymers is closer, which is not favorable for the formation of strip-pattern-spheres.

To experimentally explore the effect of rigidity of hydrophobic segments on the self-assembled structure, we prepared polystyrene-*block*-poly(ethylene glycol) (PS_15000_-*b*-PEG_5000_) coil-coil block copolymers. In contrast to PBLG, PS is a hydrophobic coil polymer. The morphology of aggregates self-assembled from PS-*b*-PEG/PS is shown in Figure S10c. Only spheres without pattern structure can be observed in the SEM characterization, which indicates rigid PBLG block plays a key role in the formation of striped patterns. The small spheres with diameters less than 50 nm are believed to be the aggregates formed by PS-*b*-PEG block copolymers.

**
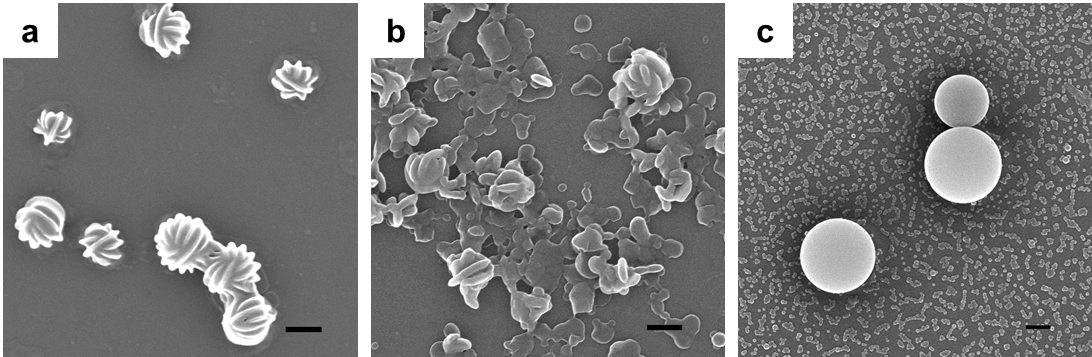
**

**Figure S10. Effects of molecular weight of PBLG blocks and rigidity of hydrophobic segments on the aggregate structures.** SEM image of aggregates self-assembled from (a) PBLG_31000_-*b*-PEG_5000_/PS_19400_; (b) PBLG_56000_-*b*-PEG_5000_/PS_19400_; (c) PS_15000_-*b*-PEG_5000_/PS_19400_. The self-assembly was performed at 20^o^C. The weight fraction of PBLG-*b*-PEG (*f*_PBLG-_*_b_*_-PEG_) is 0.5. The initial solvent is THF/DMF = 1/1 in volume. Scale bars: 400 nm.

**References**

## S1 Lin, J., Abe, A., Furuya, H. & Okamoto, S. Liquid crystal formation coupled with the coil-helix transition in the ternary system poly(γ-benzyl L-glutamate)/dichloroacetic acid/dichloroethane. *Macromolecules* 29, 2584-2589 (1996).

## S2 Lin, J., Liu, N., Chen, J. & Zhou, D. Conformational changes coupled with the isotropic–anisotropic transition part 1. Experimental phenomena and theoretical considerations. *Polymer* 41, 6189-6194 (2000).

## S3 Ding, W., Lin, S., Lin, J. & Zhang, L. Effect of chain conformational change on micelle structures:  Experimental studies and molecular dynamics simulations. *J. Phys. Chem. B* 112, 776-783 (2007).

## S4 Cai, C., Zhu, W., Chen, T., Lin, J. & Tian, X. Synthesis and self-assembly behavior of amphiphilic polypeptide-based brush-coil block copolymers. *J. Polym. Sci., Part A: Polym. Chem.* 47, 5967-5978 (2009).

## S5 Matyjaszewski, K. & Xia, J. Atom transfer radical polymerization. *Chem. Rev.* 101, 2921-2990 (2001).

## S6 Dimitrov, I. V., Berlinova, I. V., Iliev, P. V. & Vladimirov, N. G. Controlled synthesis of peptide-based amphiphilic copolymers. *Macromolecules* 41, 1045-1049 (2008).

## S7 Cai, C. *et al.* Simulation-assisted self-assembly of multicomponent polymers into hierarchical assemblies with varied morphologies. *Angew. Chem. Int. Ed.* 52, 7732-7736 (2013).

## S8 Zhuang, Z., Zhu, X., Cai, C., Lin, J. & Wang, L. Self-assembly of a mixture system containing polypeptide graft and block copolymers: Experimental studies and self-consistent field theory simulations. *J. Phys. Chem. B* 116, 10125-10134 (2012).

## S9 Wang, Y. *et al.* Self-assembly and photo-responsive behavior of novel ABC_2_-type block copolymers containing azobenzene moieties. *Soft Matter* 8, 3131-3138 (2012).

## S10 Hoogerbrugge, P. & Koelman, J. Simulating microscopic hydrodynamic phenomena with dissipative particle dynamics. *Europhys. Lett.* 19, 155-163 (1992).

## S11 Koelman, J. & Hoogerbrugge, P. Dynamic simulations of hard-sphere suspensions under steady shear. *Europhys. Lett.* 21, 363-372 (1993).

## S12 Groot, R. D. & Warren, P. B. Dissipative particle dynamics: Bridging the gap between atomistic and mesoscopic simulation. *J. Chem. Phys.* 107, 4423-4435 (1997).

## S13 Hardin, R. H., Sloane, N. J. A. & Smith, W. D. *Tables of Spherical Codes with Icosahedral Symmetry*, <http://NeilSloane.com/icosahedral.codes/.> (2014).

## S14 Miller, O. M. Notes on a cylindrical world map projection. *Geograph. Rev.* 32, 424-430 (1942).

## S15 Cai, C., Wang, L., Lin, J. & Zhang, X. Morphology transformation of hybrid micelles self-assembled from rod-coil block copolymer and nanoparticles. *Langmuir* 28, 4515-4524 (2012).

## S16 De Gennes, P.-G. & Prost, J. *The Physics of Liquid Crystals* (Clarendon press Oxford, 1993).

## S17 Kim, S. O. *et al.* Defect structure in thin films of a lamellar block copolymer self-assembled on neutral homogeneous and chemically nanopatterned surfaces. *Macromolecules* 39, 5466-5470 (2006).

## S18 Harrison, C. Mechanisms of ordering in striped patterns. *Science* 290, 1558-1560 (2000).

## S19 Horvat, A., Sevink, G. J. A., Zvelindovsky, A. V., Krekhov, A. & Tsarkova, L. Specific features of defect structure and dynamics in the cylinder phase of block copolymers. *ACS Nano* 2, 1143-1152 (2008).

## S20 Bulmer, M. G. *Principles of Statistics* (Courier Corporation, 2012).

## S21 Sawyer, L., Grubb, D. & Meyers, G. F. *Polymer Microscopy* (Springer, 2008).
